# Nasolabial Cyst

**DOI:** 10.1155/2009/586201

**Published:** 2009-10-11

**Authors:** Caner Sahin

**Affiliations:** ENT Clinic Kecioren Hospital, Akat Street 3/7 Cebeci, Ankara, Turkey

## Abstract

Nasolabial cyst is a rare nonodontogenics, soft-tissue cyst occurring in the sublabial area and anterior maxillary region. The patient usually presents with a slowly enlarging asymptomatic swelling. They are usually diagnosed in early stages because of cosmetic problems. In our paper we report a *nasolabial cyst* of a 53-year-old man and discuss the diagnosis, differential diagnosis, and treatment in the light of the literature.

## 1. Introduction

Nasolabial cysts are very rare nonodontogenic soft-tissue lesions of nasal vestibule, fossa canina, and sublabial region. The lesions cause painless swelling in sublabial fold, lips, face and cause nose obstruction. Pain can occur if the cyst becomes infected. The incidence of the cyst is 0.7% in overall chin cysts [1]. The initial diagnosis, and treatment is usually made in early stages because the lesion causes cosmetic problems; very rarely it becomes large in dimensions. 

 Herein we present diagnosis, differential diagnosis and treatment of a nasolabial cyst in the lights of the literature.

## 2. Case Report

A 53-year-old man was admitted to the ENT Department. He complained of a firm mass in the right nasolabial area that expands lips outwards. He had a history of having tooth extraction a month ago. The past medical history was unremarkable. 

**Figure 1:**
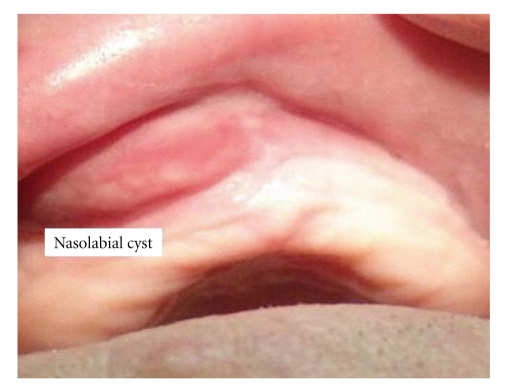
Preoperative view of the lesion in nasolabial fold.

**Figure 2:**
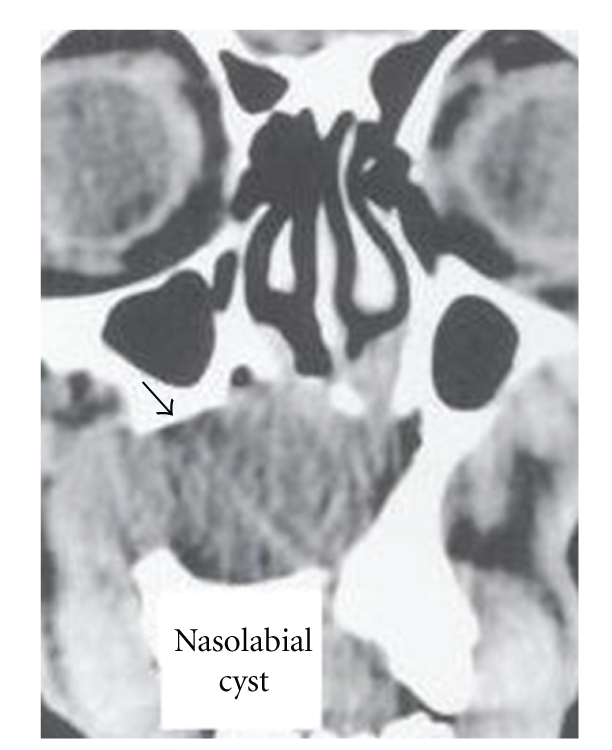
CT scan of the lesion (dark arrow indicates the lesion).

 On otolaryngologic examination, palpation revealed a swollen area corresponding to the anatomic location of the nasolabial fold. The swollen area was palpated intraorally, it was firm and nontender. The floor of nose was swollen and narrowed by a mass from the inferior side. The lesion was 2 × 3 cm in dimensions (Figure 1). The CT scan revealed a nonodontogenic cyst in nasolabial area (Figure 2). Findings on blood and serum biochemistry were within normal limits.

The nasolabial cyst was excised via a sublabial intraoral incision under general anesthesia. The cyst was dissected free, the wound was closed in layers. There were no postoperative complications. The histopathological examination revealed nasolabial cyst. The symptoms resolved after the operation.

## 3. Discussion

Nasolabial cysts were first described by Zuchercandl in 1892 [2]. They are nonodontogenic masses that can be seen in the maxillofacial area. In the literature, the lesions are named as nasolabial cyst, nasoalveolar cyst and Klesdath tumour [2]. The lesion is submucosal and extraosseous, it expands via the gingivobuccal sulcus and expands all the soft-tissues outwards. Usually the cysts are seen in the 4th-5th decade of lifetime. The incidence of bilateral cyst is 10% in the literature [3].

There are three theories for the formation of the cyst.

1. The cyst is formed embryologically by detention cells in the maxilla, medial, and lateral nasal wall.
2. The cyst is formed embryologically by detention cells from the inferior nasolacrimal channel redundant cells.
3. The cyst is formed embryologically by detention cells from the inferior nasolacrimal channel endodermal cells [4]. Exposure to trauma accelerates the formation of the cyst [4].

The differential diagnosis of the cyst must be made with central line cysts, cyst of maxilla, odontogenic cysts, periapical cysts, periapical abscess, periapical granulomas, epidermal inclusion cyst, frunculosis of *base of* the nose, and neoplasms of base of the nose [5]. The safety of the tooths in the nasolabial region is clinically important in differentiating from the other lesions. Radiological examination is important in differential diagnosis of odontogenic and nonodontogenic cysts of the region. We expect no erosion of bone especially in the early stages of the disease [6]. The diagnosis of the lesion can be made by clinical, radiologic *examination* and histopathological examination. 

 The treatment can be made by surgical exicion, injection of sclerozing materials in the cyst, and endoscopic marsupialization methods [7]. Exicion of the cyst via the sublabial incision is the most preferred treatment modality with very low recurrence rate and cosmetic reasons. Sublabial incision is much beter than external incision especially in terms of cosetic reasons. Recurrence doesnot happen if the wall of the sac is competely removed. There is a reported case of malignant degeneration of the cyst in the literature [8]. The nasolabial cysts can be marsupialized transnasally under the guidance of nasal endoscopes [9]. 

 Nasolabial cyst must be kept in mind in differential diagnosis of nasal vestibule, nasal base, and sublabial area [10]. The diagnosis and treatment by surgical exicion is simple but there has to be made a differantial diagnosis with odontogenic, nonodontogenic cysts of the region. Herein we present a case of a nasolabial cyst and discuss it in the lights of the literature.

## References

[1] El-Din K, el-Hamd AA (1999). Nasolabial cyst: a report of eight cases and a review of the literature. *Journal of Laryngology and Otology*.

[2] Kuriloff DB (1987). The nasolabial cyst-nasal hamartoma. *Otolaryngology—Head and Neck Surgery*.

[3] Barzilai M (1994). Case report: bilateral nasoalveolar cysts. *Clinical Radiology*.

[4] Nixdorf DR, Peters E, Lung KE (2003). Clinical presentation and differential diagnosis of nasolabial cyst. *Journal of the Canadian Dental Association*.

[5] Choi JH, Cho JH, Kang HJ (2002). Nasolabial cyst: a retrospective analysis of 18 cases. *Ear, Nose and Throat Journal*.

[6] Hashida T, Usui M (2000). CT image of nasoalveolar cyst. *British Journal of Oral and Maxillofacial Surgery*.

[7] Su C-Y, Chien C-Y, Hwang C-F (1999). A new transnasal approach to endoscopic marsupialization of the nasolabial cyst. *Laryngoscope*.

[8] López-Ríos F, Lassaletta-Atienza L, Domingo-Carrasco C, Martinez-Tello FJ (1997). Nasolabial cyst: report of a case with extensive apocrine change. *Oral Surgery, Oral Medicine, Oral Pathology, Oral Radiology, and Endodontics*.

[9] Jae Yong L, Byoung Joon B, Jang Yul B, Hyuck Soon C, Byung Don L, Dong Wook K (2009). Comparison of conventional excision via a sublabial approach and transnasal marsupialization for the treatment of nasolabial cysts: a prospective randomized study. *Clinical and Experimental Otolaryngology*.

[10] Choi JH, Cho JH, Kang HJ (2002). Nasolabial cyst: a retrospective analysis of 18 cases. *Ear, Nose and Throat Journal*.

